# Eveningness chronotype and depressive affective temperament associated with higher high-sensitivity C-Reactive Protein in Unipolar and Bipolar Depression

**DOI:** 10.1192/j.eurpsy.2023.566

**Published:** 2023-07-19

**Authors:** L. Orsolini, S. Pompili, A. Cicolini, L. Ricci, U. Volpe

**Affiliations:** Unit of Clinical Psychiatry, Department of Neurosceinces/DIMSC, Università Politecnica delle Marche, Ancona, Italy

## Abstract

**Introduction:**

Several studies investigated the role of inflammation in the etiopathogenesis of psychiatric disorders, by also evaluating how CRP may exert a pathoplastic and/or psychopatological role in mood disorders.

**Objectives:**

The aim of our cross-sectional study is evaluating the high-sensitivity C-reactive-protein (hsCRP) levels in a cohort of unipolar and bipolar depressive inpatients, in relation with psychopathological, temperamental and chronotype features.

**Methods:**

Among 313 screened inpatients, we recruited 133 moderate-to-severe depressive patients who were assessed for hsCRP levels, chronotype with Morningness-Eveningness Questionnaire (MEQ) and affective temperament with Temperament Evaluation of Memphis, Pisa, Paris and San Diego (TEMPS).

**Results:**

hsCRP levels were significantly higher among those with previous suicide attempt (p=0.05), death (p=0.018) and self-harm/self-injury thoughts (p=0.011). In addition, hsCRP levels were significantly higher among patients with hypertension (p=0.020) and dyslipidemia (p=0.013). Moreover, positive correlation were found between hsCRP levels and the number of illness of years (p<0.001). Significant positive correlation were found between hsCRP levels and depressive (p<0.001) and cyclothymic (p<0.001) affective temperaments, while a negative correlations were reported between hsCRP levels and hypertimic (p<0.001) and irritable (p=0.029) affective temperaments. Eveningness chronotypes subject displayed higher hsCRP levels compared to intermediate-type and morningness-type chronotypes (p<0.001). Linear regression analyses, adjusted for all covariates, demonstrated that higher scores at the TEMPS-M depressive, while lower scores at the hyperthymic and irritable affective temperaments [F=88.955, R^2^=0.710, p<0.001] and lower MEQ scores [F=75.456, R^2^=0.405, p<0.001] statistically significantly predicted higher hsCRP.

**Conclusions:**

Eveningness chronotype and a depressive affective temperament appeared to be associated with higher hsCRP levels during moderate-to-severe unipolar and bipolar depression. Further longitudinal and larger studies should better characterise patients with mood disorders by investigating the influence of chronotype and temperament.

**Disclosure of Interest:**

None Declared

